# Diagnosis Wanted

**Published:** 1893-11

**Authors:** S. H. Barham

**Affiliations:** Caledonia, Texas


### DIAGNOSIS WANTED.

CALEDONIA, TEXAS, Oct. 9, 1893.

*Editor Texas Medical Journal:*

The following case presents to me some features, so unlike anything heretofore occurring, in a moderately extensive practice, as well as a somewhat varied experience with worms among children, that I am induced to report it more with the hope of gaining information from some one whose information and experience have been more varied and extensive than my own, rather than with any idea of adding anything to the literature on the subject:

E. H., male, age 17, bottle-fed in infancy, delicate in organization, but having no previous history of organic disease, or protracted sickness, sent to my office, about a year ago, three small worms, the largest of which was about one-half an inch in length, and which had passed from his bowels on the day before. At this time they had been passing, at intervals of a few weeks, for about eighteen months, at times only few in numbers, at other times many; but the patient being of well-to-do parentage, was of a modest and retiring disposition, and the circumstance was so mortifying to his sensitive and refined nature, that he withheld the knowledge from his parents, or any one, and could not be induced or pursuaded to apply for relief. At this time the father became cognizant of the trouble, and brought me this specimen, which retained life four days in a tightly-corked bottle. No specimen has been detected larger than half an inch, and they range downward to the smallest point.

To the unaided eye they appear of white color, flattened on the under side, with a gradual taper from the caudal extremity to the head, which is a small black spot. They are of sluggish motion, and have the appearance of moving "tail foremost," or else the head is on the wrong end.

My attention had never been called to anything of the kind before, but having since had an opportunity of examining the maggot of the common green fly, they appear to be identical with it in every particular, except the more slugglish motion of the former.

Having had the patient under my observation and treatment about a year, I notice an interval of about three weeks in the passage of the worms, independent of whatever germicide he may be taking. Indeed, I have noted the evacuation of a lot of them a few times, a week following the administration of a course of santonine, with the entire absence of worms in the discharges during the administration, and in each instance this evacuation would correspond with the periodic interval of three weeks.

I have no disposition to lengthen this article with the details of the treatment; it is needless to do so. Let it suffice that the treatment for worms in general, as laid down by the authors of latest works, has been followed; and I have rung the changes on about all the various parasiticides known to our pharmacopœia, alone and variously combined with general tonics, and with only a partial benefit to the patient's general health, and the absence of any fully-developed worms for the past six months. The patient reports them passing with about the same regularity, but all of small size.

The case is reported with the hope that it will elicit a reply from some one of your many readers whose observation of parasites has been more varied than the writer's, or from some who have made specialties of parasitic diseases.

I will only remark in closing that there is an entire absence of anal irritation, a leading symptom of a habitat in the rectum. But where is its habitat, and what is its species? are questions I cannot solve from the literature at my command. I submit it with the hope that some of the readers of the JOURNAL may give some information of its species and its habitat, and suggest a line of treatment for its complete expulsion and thorough eradication from the system. S. H. BARHAM, M. D.

